# The Effect of Virtual Reality on the Reduction of Pain in Women with an Indication for Outpatient Diagnostic Hysteroscopy: A Randomized Controlled Trial

**DOI:** 10.3390/jcm12113645

**Published:** 2023-05-24

**Authors:** Jesus A. Pelazas-Hernández, David Varillas-Delgado, Teresa González-Casado, Ignacio Cristóbal-Quevedo, Agustina Alonso-Bermejo, Marina Ronchas-Martínez, Ignacio Cristóbal-García

**Affiliations:** 1 Department of Obstetrics and Gynaecology, El Escorial University Hospital, 28200 Madrid, Spain; jesuspelazas@gmail.com (J.A.P.-H.); agustina.alonso@salud.madrid.org (A.A.-B.); marina.ronchas@gmail.com (M.R.-M.); 2 Faculty of Medicine, Universidad Francisco de Vitoria, 28223 Madrid, Spain; ignacio.cristobal@salud.madrid.org; 3 Faculty of Health Sciences, Universidad Francisco de Vitoria, 28223 Madrid, Spain; 4 Department of Obstetrics and Gynaecology, Sierra de Guadarrama Health Centre, 28440 Madrid, Spain; tglezcasado@hotmail.com; 5 Department of Obstetrics and Gynaecology, La Paz University Hospital, 28046 Madrid, Spain; ignaciocristobal94@gmail.com; 6 Department of Obstetrics and Gynaecology, San Carlos Clinic Hospital, 28040 Madrid, Spain

**Keywords:** hysteroscopy, outpatients, virtual reality, visual analogue scale, pain perception

## Abstract

*Background:* The cognitive distraction caused by Virtual Reality (VR) seems to cause a decrease both in pain and its perception as in the time spent thinking about possible pain, among anxiety about hysteroscopy procedure. The main objective of this investigation was to evaluate the efficacy of virtual reality for pain relief during outpatient hysteroscopy. *Method*: A total of 83 patients underwent outpatient diagnostic hysteroscopy in a single-centre, open-label, randomized control trial. Overall, 180 women with medical indication for an outpatient diagnostic hysteroscopy were randomized. Ten were excluded due to the impossibility of entering the endometrial cavity caused by a cervical canal that was not permeable, and 15 did not tolerate the pain at the beginning and during the procedure, excluding themselves from the final model. Finally, 154 were analysed per protocol to use VR (*n* = 82, study group) or standard treatment (*n* = 72, control group) assessing the differences between both groups by reduction in pain using Visual Analogue Scale score (VAS: 0–10 cm) and clinical data (arterial pressure, heart rate, and oxygen saturation) at the end of hysteroscopy, at 15 and 30 min after hysteroscopy. *Results*: Women with VR outpatient diagnostic hysteroscopy experienced less pain at final (VAS score 2.451 vs. 3.972, standard mean difference (SMD) −1.521, 95% CI −2.601 to −0.440; *p* = 0.006), at 15 min (VAS 1.769 vs. 3.300, SMD −1.531, 95% CI −2.557 to −0.504; *p* = 0.004), and at 30 min (VAS 1.621 vs. 2.719, SMD −1.099, 95% CI −2.166 to −0.031; *p* = 0.044) after the ending of the hysteroscopy, compared with no VR. *Conclusions*: The use of VR during outpatient diagnostic hysteroscopy proved effective in the reduction of pain in this randomized control trial. It shows wide potential role in ambulatory gynaecologic procedures to avoid repeating tests, perform surgeries without anaesthesia, and the use of medication and its side effects.

## 1. Introduction

The appearance of hsysteroscopes with smaller calibres allows for a minimally invasive approach and can be performed on an ambulatory basis [1] as a safe, fast, and economically profitable technique [2]. It is not free of complications such as uterine trauma (lacerations or perforations) or infections, but the perception of pain is the most relevant complication and the main reason for the failure of the procedure [3,4].

Multiple treatments have been used to try to alleviate the pain, objectifying that the use of analgesia (oral, topic, opioids, injected local anaesthesia, cervical nerve block, etc.) along with an adequate prior cervix preparation does not provide a consistent adequate quality evidence on the safety nor the effectiveness of any treatment in this group of patients, with the current recommendation being not to use analgesics or the prior cervix preparation in a routinely approach ahead of the procedure, as they can cause side effects such as bleeding, blood clots in the legs or pelvis, infections, and uterine perforation, without reducing pain during the hysteroscopy [5,6,7]. Outpatient hysteroscopy is a technique increasingly used in the diagnosis and even treatment of cervical and endometrial pathology [8]. There is unanimity that the use of smaller calibre hysteroscopies, insufflation pressures less than 90–100 mmHg, and the vaginoscopy approach reduce the pain reported with the test, but there is not a single criteria on the use of drugs for cervical preparation, misoprostol type, or the type of analgesics to be used, non-steroidal anti-inflammatory drugs (NSAIDs), opioids, antispasmodics, etc., both for their effectiveness and for their side effects [9].

Virtual reality (VR), a relatively new intervention, has been studied as a distraction technique for nonpharmacological pain relief [10]. Put simply, it is a computer-generated representation of an immersive environment viewed through a headset [11]. The cost, quality, and accessibility of VR devices has significantly improved in recent years and has offered novel application in the medical field. Virtual reality for managing pain has been studied in paediatrics, dentistry, burns treatment, chronic pain, labour, episiotomy, and phobias [12,13,14,15]. Although a meta-analysis suggested that VR may have a role in reducing pain scores in acutely painful procedures, it was found to be effective only in needles and burns physical therapy [16]. The studies of VR on pain and anxiety however were limited by clinical and statistical heterogeneity [16,17]. It has been observed that VR can lead to cognitive distractions due to the subjective psychological illusion of the world recreated in the minds of individuals undergoing VR and neurobiology studies have also noticed a decrease in the brain’s activity associated with pain using VR [1,5,9]. This cognitive distraction seems to cause a decrease both in pain and its perception, such as in the time spent thinking about possible pain as well as anxiety about the procedure [1,5].

Findings in a randomised controlled trial in paediatric cancer patients undergoing peripheral intravenous cannulation procedure indicate that VR is safe and effective to alleviate pain and anxiety in this population [18]. Most of these new studies that present the advantage of using VR for pain relief in these specialties are randomised controlled trials. However, a recent narrative review showed that the VR exposure groups had a worsening of acute pain scores, without providing relevant results favouring the use of virtual techniques [19]. In addition, it has been observed that the results of the use of VR versus analgesics type NSAIDs (ibuprofen, ketorolac, celecoxib, etc.), opioids tramadol type, antispasmodics (drotaverine), misoprostol, inhalation of NO_2_, paracervical nerve blocks, TENS (Transcutaneous Electrical Nerve Stimulation) presents at least the same results as the best ones regarding the reduction in pain perception [20,21] with few or no side effects. VR holds promise as a nonpharmacologic analgesic and anxiolytic intervention for otolaryngology office-based procedures, represents a nonpharmacologic option for pain relief in pregnant or labouring women, and is associated with a reduction in pain in nulliparous women in labour [22]. It is therefore an acceptable option to help manage medical trauma during infusions in children and youth with special heath care needs [23].

Therefore, the aim of this study was to assess whether patients with a medical indication for outpatient diagnostic hysteroscopy benefit from the use of VR to decrease the pain perception compared to current clinical practice standard treatment of non-use analgesia.

## 2. Materials and Methods

### 2.1. Patients

This is a single-centre, open label, randomized controlled trial. The protocol was approved by the Clinical Research Ethics Committee, Puerta de Hierro University Hospital, Madrid (IRB code, 12.18) and conducted in El Escorial University Hospital, Madrid, Spain. It was registered with ClinicalTrials.gov (accessed on 24 January 2019) (NCT03827824) prior to enrolment. Between March 2019 and October 2019, consecutive women with an indication for outpatient diagnostic hysteroscopy according to the criteria of *the Sociedad Española de Ginecología y Obstetricia* (SEGO, Spanish Society Obstetrics and Gynaecology) were invited to participate in the trial. Only data on pain perception among patients with VR and standard treatment are presented in the manuscript, with a deviation of the study protocol in which data on anxiety and pain perception are presented.

All patients agreed to participate in the study and signed a written informed consent. Women were randomly allocated after baseline data collection in a parallel-group (1:1 ratio) to either a VR (study group) or standard treatment (control group). Participants were randomized on an Excel-generated randomization schedule, without block randomisation. The research coordinators regularly performed data quality control, management, and protocol compliance verification. Due to the nature of the intervention, blinding of participants, care providers, and outcome assessors was not possible.

Inclusion criteria were: (i) those established by SEGO [1] to perform an hysteroscopy in the physician’s office; heavy menstrual bleeding (HMB), suspected endometrial pathology (hyperplasia, polyps, myomas, etc.), postmenopausal bleeding and removal of a foreign body (IUD, etc.); (ii) ages between 18 and 75 years; (iii) understand the study’s characteristics, being able to make the voluntary decision to participate and sign the consent after being correctly informed. Exclusion criteria included: (i) the use of some type of contraceptive (oral, vaginal, intrauterine, dermal, or subcutaneous) because they would confound the study; (ii) previous hysteroscopy; (iii) the use of any type of analgesic for other pathologies; and (iv) presenting any type of disease or disability that may intervene in the aim of this study (any visual, auditory or sensory deficits, diseases or mental syndromes that could make the study difficult or impossible to carry to completion). An a priori sample size calculation indicated that patients of one gynaecology hospital service were needed to obtain statistically significant differences between VR and standard treatment. This a priori sample size was calculated to obtain an effect size of 23.8% of reduction in pain (statistical power of 80% with type I error set at 5%), based on a previous investigation that obtained these results when they study the effect of intervention regarding control group in pain relief [24]. A target sample size of 150 patients was determined using G*Power software, v.3.1.9.7 [25].

The study methods have been reported in accordance with the CONSORT statement (Supplementary Materials).

### 2.2. Intervention

The patients were selected by different physicians from the gynaecology’s office, where they were requested an appointment for an outpatient diagnostic hysteroscopy following the criteria of the SEGO and priority established by Madrid Region, oncological priority, preferential, and non-priority. The administrative staff of the Hospital Admission Service provided them with a day and a serial number for their appointment. On the day, the signed consents were collected and any possible doubts were answered by the gynaecologist who would perform the procedure. At that time and based on their appointment order generated by admission, the patients were included in the different groups—the study group or the control group—according to the appointment order and the corresponding number obtained using the stipulated randomisation computer program.

All the hysteroscopies were performed by the same gynaecologists, in the same room, and with the same equipment [26].

All procedures were performed with a rigid 5 mm diagnostic hysteroscope, Bettocchi model. All surgical instruments that were used when necessary were semi-rigid, 5 Charr., with a length of 34 cm. The vaginoscopy approach was used to access the endometrial cavity [27,28]. Distension media: physiological saline solution, using an electronic perfusion pump with initial irrigation pressures of 75 mmHg up to a maximum of 85 mmHg [29].

Patients included in the intervention group were placed with the VR glasses, Samsung’s Oculus GO model. This model is equipped with spatial sound drivers built into the viewfinder, so no external headphones are required or used.

The chosen distraction was a free demo called “A Night Sky, where the patient is invited to relax on the edge of a peaceful vale, beside a crackling campfire, and beneath the open night sky—connecting constellations to form patterns among the stars using a device “go controller” in the dominant hand as a “laser pointer”. As the different shapes are revealed, wonderful creatures are brought to life”.

A similar procedure was carried out with the standard treatment patients in the control group.

### 2.3. Measurement Variables

The primary outcome was the pain score during the hysteroscopy, repeated 15 min and 30 min after the end of the procedure measured on a Visual Analogue Scale (VAS: 0–10 cm) [30] at rest as the average pain over the last 24 h, with 0 indicating “no pain” and 10 “worst pain imaginable”. VAS has demonstrated to be reliable, valid, and responsive in assessing the treatment outcome in persons with patellofemoral pain [31]. Secondary outcomes were systolic arterial pressure (SAP) and diastolic arterial pressure (DAP), compiled in millimetres of mercury as a measurement unit and divided into SAP and DAP. Heart rate and oxygen saturation during hysteroscopy, 15 min, and 30 min after the end of the procedure were measured by a pulse oximeter placed on the index finger of the non-dominant hand, according to the existing protocols [32]. Blood pressure measurements were performed, in a lithotomy position and with the cuff on the left arm, prior to performing hysteroscopy, when passing through the Internal Cervical Os (OCI) and at the end of the test.

### 2.4. Statistical Analysis

Continuous data were summarised as mean and standard deviation, and categorical data as counts and percentages. Demographic data were compared using the chi-square test for qualitative variables and *t*-test for quantitative variables. Between group differences were reported with 95% confidence intervals (95% CI) and *p*-value (using *t*-test to compare normally distributed data). Assuming a power of 80% to detect difference in pain scores between groups with two-tailed at 95% CI, the standardized mean difference (SMD) was used for measuring the pain-effect. Cohen’s d, the difference in scores measured on a standard deviation scale, was used to determine the effect size, with values above 0.7 considered to be large [33]. We used a mixed-model repeated measurements analysis of variance (RMANOVA) with patients as a random factor (repeated measurements at end, 15 min, and 30 min after hysteroscopy), the baseline value as a covariate, and assuming a covariance structure with compound symmetry. Statistical analysis was performed per protocol (PP) using SPSS 21.0^®^ software (IBM Corp. Released 2012. IBM SPSS Statistics for Windows, Version 21.0. Armonk, NY, USA: IBM Corp).

## 3. Results

**Figure 1:**
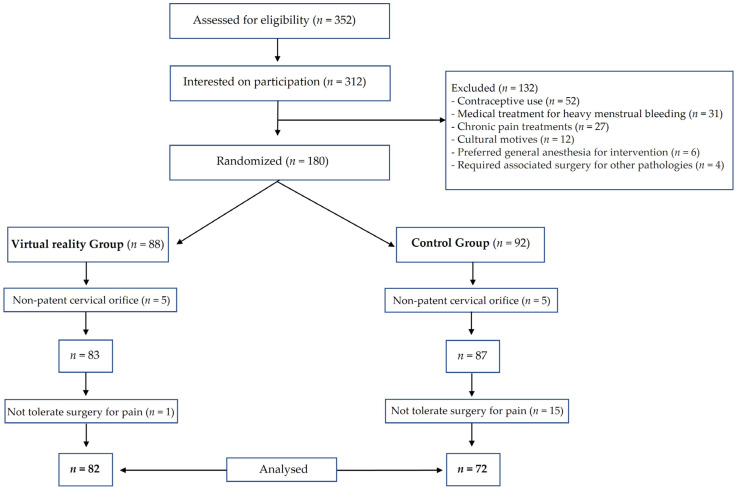
Flowchart.

From March to October 2019, 352 women with an indication for outpatient diagnostic hysteroscopy according to the criteria of SEGO were selected from the El Escorial University Hospital gynaecologist’s office. A total of 312 women were interested to participate. Out of this number, 52 had a contraceptive treatment and 80 did not meet the eligibility criteria. A total of 180 women were finally randomised into 2 groups: 88 in the study group and 92 in the control group. Ten were excluded due to the impossibility of entering the endometrial cavity caused by a cervical canal that was not permeable, which included five in the study group and five in the control group. Fifteen women in the control group and one in the study group did not tolerate the pain at the beginning and during the procedure and were cited for surgical hysteroscopy under anaesthesia, excluding themselves from the final model. Finally, 154 women were analysed PP: 82 in study group and 72 in control group (Figure 1).

### 3.1. Baseline Characteristics

Baseline characteristics were similar in both groups regarding age, reproductive stage, parity, smoking habit, previous surgery on the cervix, biopsy sampling, performing polypectomy, or myomectomy during the hysteroscopy (Table 1).

### 3.2. Main Outcomes

Only 10 women in the study have had previous surgeries on the cervix, 23 were nulliparous, and 61 were experiencing menopause (Table 1).

The suspension rate due to pain during hysteroscopy was 6.9% in the control group versus 6.1% in the study group.

The procedure time was 6.67 min (±0.52 min) in the study group and 7.95 min (±1.01 min) in the control group. The time for VR goggles placement and resolution of the patients’ doubts was 10.94 min (±0.95 min) in the study group and 10.34 min (±1.42 min) in the control group.

The VR intervention showed a statistically significant reduction in pain at the end of the hysteroscopy (*p* = 0.006), at 15 min (*p* = 0.004) and 30 min after surgery (*p* = 0.044) compared to the standard treatment (Table 2).

There was a significant effect of group (F = 5.604; *p* = 0.024) on VAS. There was also a significant effect of time (F = 10.175; *p* < 0.001). However, time x group interaction was not significant (F = 0.945; *p* = 0.394).

### 3.3. Physiological Outcomes

Physiological changes in blood measurements showed no statistical results between groups before hysteroscopy on heart rate (*p* = 0.526), SatO_2_ (*p* = 0.797), and blood pressure (*p* = 0.558). During procedure; heart rate (*p* = 0.237), SatO_2_ (*p* = 0.732), and blood pressure (*p* = 0.584) and at the end of hysteroscopy; heart rate (*p* = 0.802), SatO_2_ (*p* = 0.633), and blood pressure (*p* = 0.293) (Table 3).

Four cases of vagal reaction have been described in our study (2.6%), specifically dizziness during the hysteroscopy, of which there were three in the control group (3.6%) (two women underwent biopsy) and one in the study group (1.4%) (a biopsy was performed). No woman in the study required any treatment except for posture changes.

## 4. Discussion

In this randomised controlled trial, we have been able to observe that the virtual reality pain management intervention had a large effect in reducing the pain in outpatient diagnostic hysteroscopy at the end of the procedure, at 15 and 30 min after surgery, and similar values were shown in both groups regarding blood measurements and SatO_2_, but with a greater in the virtual reality group compared to the control group. Among the registered side effects, four minor reflex syncope stand out, of which there were three in the control group versus one in the virtual reality group.

Compared to standard treatment, the virtual reality pain management intervention had a large effect in reducing pain in outpatient diagnostic hysteroscopy. This effect was robust, even after controlling for baseline pain expectations and a range of patient covariates. Staff and the majority of patients found the procedure to be both feasible and acceptable and patients reported a range of experiences, suggestive of the mechanisms by which VR technology may influence pain via immersion, relaxation, distraction, and imagery. The study additionally demonstrated the willingness of patients to participate. Insights generated from the themes suggested offering a multimodal pain relief strategy to improve the experience at outpatient hysteroscopy. Quantitative analysis suggested patient profiling based on history, taking into consideration patient preferences by offering a variety of distraction techniques with a range of videos to choose from if they were to choose virtual reality as a distraction technique.

The analysis offered key insights into patient expectations concerning the degree of pain relief that is possible with virtual reality technology and implementation strategies to facilitate around the transfer of research finding in a clinical setting. 

To avoid bias, the same diversion, A Night Sky, which is a free version included in the Oculus Go catalogue, was used on all patients. The authors believe that by using a VR option that could be chosen according to the patient’s taste, with greater immersion possibilities, the results presented in this trial would be even more significant. Subsequent works should look at the type of VR technology and anxiety perception, the context where it is deployed and for what kind of procedure. The optimal VR program may vary according to patient characteristics, the invasiveness of the intervention, and its duration. VR technology should be tested in more painful gynaecological interventions such as endometrial ablation, cervical biopsy, and transvaginal egg collection. Moreover, the prospective benefit of VR does not need to be restricted to gynaecological practice but should be evaluated in a whole host of ambulatory procedures involving conscious patients [34].

Side effects have been described during hysteroscopy performed by a gynaecologist [4], which are commonly related to vagal reactions (nausea, vomiting, dizziness, sweating, vertigo, bradycardia, and hypotension) or to the use of different methods used to reduce pain such as drowsiness linked to opioids, back pain with TENS, epigastralgia, gastritis, and skin rash linked to the NSAIDs. However, it also represents not only an improvement for women but also an improvement for the health system by avoiding having to duplicate tests, perform surgeries with anaesthesia, the use of medication, as well as for the gynaecologist, as the persistence of symptoms after 30 min from the end of the hysteroscopy is practically nil, as well as the described side effects in previous studies [35,36].

During hysteroscopy, a moderate to severe pain level has been reported, which is defined as a score of 4 or higher on a VAS scale of 10 cm [5]. The role of VR on pain management has been previously published in a wide variety of specialties and settings; recently in women with an indication for outpatient diagnostic hysteroscopy [10,34], showing an impressive preliminary result. Reduction in peri-procedural pain was statistically significant but more importantly the effect size of a 2 cm (20%) difference on a 10-cm visual analogue scale must be clinically significant, and similar results shown in this work (ES 1.46 to 1.99 cm (14.6 to 19.9%) difference) favour the study group. These results are similar to the recently published randomised controlled trial by Deo et al. [10], which found that the use of VR significantly decreased pain during outpatient hysteroscopy by 28%. In turn, these data are similar to the randomized controlled trial by Wong et al. [22], which found that the use of virtual reality reduces perceived pain by 23.8% compared to standard treatment. Another recent randomized controlled trial by Fouks et al. [37] shows that the use of virtual reality does not decrease pain in patients during hysteroscopy, but helps to decrease heart rate, which is contradictory to the data shown in this research. These previous studies have a significantly smaller sample size than the one presented—44, 40, and 82 women, respectively [10,22,37], and these results are less biased than previous studies. Therefore, future studies are needed to confirm the findings presented in this research and thus to correct the controversy that still exists in this field of research.

The greatest strength of our study resides in being a randomised controlled study, where the selection of the subjects was made from the doctor’s office by different gynaecologists not associated with the office of hysteroscopy by the SEGO criteria and the inclusion in the different study groups was made by a program using an agenda created by an Administrative Assistant from outside the Gynaecology Department, avoiding selection bias. The good results due to the application of VR are very promising and since the hardware used is financially accessible, it is possible to scale the solution for many hospitals (including public ones). This study presents VR as a potential role in outpatient gynaecological procedures in need of further evaluation. The use of VR also represents not only savings for the health system by avoiding repeat tests, surgeries without anaesthesia, the use of drugs, but also and above all, for the women by not needing to repeat tests, reducing the risks of anaesthesia and surgeries, the use of drugs and their side effects, stress, as well as loss of work or leisure hours. VR technology should be tested in more painful gynaecological interventions such as endometrial ablation cervical biopsy and transvaginal egg collection.

This study presents several limitations; first, this research did not present anxiety during hysteroscopy, a result that will be addressed in future studies to complete the evidence presented in this research; and second, other avenues would be interesting, including the use of VR in patient education to familiarise patients with the procedure and use as a triage before offering it as a pain relief intervention.

## 5. Conclusions

The use of virtual reality diversions is a well-tolerated option that decreases pain perception after outpatient diagnostic hysteroscopy in women compared to standard treatment, without side effects and with less suspension rate due to pain, reducing the need for other surgical tests with anaesthesia that would increase the risk rate for these women by proposing to use a pain killer after a hysteroscopy.

## Figures and Tables

**Table 1:** Baseline characteristics of the patients allocated to the study or control groups.

		Study Group (*n* = 82)	Control Group (*n* = 72)	*p*-Value
**Age, mean (SD)**		47.19 (8.716)	49.20 (11.789)	0.440
**SatO_2_ initial, mean (SD)**		97.68 (1.873)	97.00 (2.093)	0.139
**SAP initial, mean (SD)**		132.03 (23.037)	139.49 (29.347)	0.213
**DAP initial, mean (SD)**		79.48 (14.744)	78.71 (11.668)	0.807
**Heart Rate, mean (SD)**		83.73 (13.568)	81.49 (16.823)	0.526
**VAS, mean (SD)**		3.75 (1.134)	4.54 (1.253)	0.174
**Menopause**	**No, *n* (%)**	52 (63.4)	41 (56.9)	0.144
	**Yes, *n* (%)**	30 (36.6)	31 (43.1)	
**Parity**	**Nulliparous, *n* (%)**	14 (17.1)	9 (12.5)	0.409
	**Multiparous, *n* (%)**	68 (82.9)	63 (87.5)	
**Smokers**	**No, *n* (%)**	44 (53.6)	35 (48.6)	0.415
	**Yes, *n* (%)**	38 (46.4)	37 (51.4)	
**Previous Conization**	**No, *n* (%)**	78 (95.1)	66 (91.7)	0.585
	**Yes, *n* (%)**	4 (4.9)	6 (8.3)	
**Biopsy**	**No, *n* (%)**	82 (100.0)	70 (97.2)	0.892
	**Yes, *n* (%)**	0 (0.0)	2 (2.8)	
**Myomectomy**	**No, *n* (%)**	80 (97.6)	68 (94.4)	0.742
	**Yes, *n* (%)**	2 (2.4)	4 (5.6)	

DAP, Diastolic Arterial Pressure; SAP, Systolic Arterial Pressure; SatO_2_, Oxygen Saturation; SD, Standard Deviation; VAS, Visual Analogue Scale.

**Table 2:** Pain assessment at the end of hysteroscopy at 15 min and 30 min in the study and control groups.

	VAS				
	Study Group (*n* = 82)	Control Group (*n* = 72)	SMD (95% CI)	ES [95% CI]	*p*-Value
**Final, mean (SD)**	2.451 (1.173)	3.972 (0.519)	−1.521 (−2.601 to −0.440)	−1.797 [−3.152 to −0.321]	0.006
**15 min, mean (SD)**	1.769 (0.488)	3.300 (1.047)	−1.531 (−2.557 to −0.504)	−1.996 [−3.826 to −0.472]	0.004
**30 min, mean (SD)**	1.621 (0.383)	2.719 (1.146)	−1.099 (−2.166 to −0.031)	−1.437 [−2.942 to −0.152]	0.044

95% CI, 95% Confidence Interval; ES, Effect size; SD, Standard Deviation; SMD: Standard Mean Difference; VAS, Visual Analogue Scale.

**Table 3:** Physiological changes in the respiratory and cardiovascular system in the study and control groups.

	Initial			Procedure			Final		
	Study Group (*n* = 82)	Control Group (*n* = 72)	*p*-Value	Study Group (*n* = 82)	Control Group (*n* = 72)	*p*-Value	Study Group (*n* = 82)	Control Group (*n* = 72)	*p*-Value
**SatO_2_, mean (SD)**	97.68 (1.873)	97.00 (2.093)	0.797	98.05 (1.749)	97.11 (2.246)	0.732	98.15 (1.514)	97.17 (2.332)	0.633
**SAP, mean (SD)**	132.03 (23.037)	139.49 (29.347)	0.213	133.13 (20.756)	137.46 (17.094)	0.440	127.54 (19.316)	133.94 (24.352)	0.212
**DAP, mean (SD)**	79.48 (14.744)	78.71 (11.668)	0.807	76.85 (11.142)	77.23 (12.291)	0.889	73.56 (12.703)	76.11 (14.338)	0.420
**Blood Pressure, mean (SD)**	96.38 (16.429)	98.54 (15.257)	0.558	95.05 (13.199)	96.89 (15.479)	0.584	91.03 (13.523)	94.66 (15.961)	0.293
**Heart Rate, mean (SD)**	83.73 (13.568)	81.49 (16.823)	0.526	76.79 (14.577)	72.89 (13.486)	0.237	74.41 (11.621)	75.20 (15.262)	0.802

DAP, Diastolic Arterial Pressure; SAP, Systolic Arterial Pressure; SatO_2_, Oxygen Saturation; SD, Standard Deviation.

## Data Availability

The data presented in this study are available on request from the corresponding author. The data are not publicly available due to legal restrictions.

## Supplementary Materials

The following supporting information can be downloaded at: https://www.mdpi.com/article/10.3390/jcm12113645/s1, CONSORT 2010 checklist of information to include when reporting a randomised trial and CONSORT 2010 Flow Diagram.
